# Clinical impact of metagenomic next-generation sequencing of peripheral blood for the diagnosis of invasive mucormycosis: a single-center retrospective study

**DOI:** 10.1128/spectrum.03553-23

**Published:** 2023-12-14

**Authors:** Wei Wang, Yake Yao, Xi Li, Shanshan Zhang, Zhu Zeng, Hua Zhou, Qing Yang

**Affiliations:** 1 Department of Respiratory and Critical Care Medicine, The First Affiliated Hospital, Zhejiang University School of Medicine, Hangzhou, China; 2 Department of Respiratory and Critical Care Medicine, Shaoxing Central Hospital, Shaoxing, China; 3 Department of Clinical Laboratory, Laboratory Medicine Center, Zhejiang Provincial People’s Hospital, Affiliated People’s Hospital, Hangzhou Medical College, Hangzhou, Zhejiang, China; 4 Department of Respiratory and Critical Care Medicine, Beilun People’s Hospital, Ningbo, China; 5 Department of Clinical Laboratory, The First Affiliated Hospital, Zhejiang University School of Medicine, Hangzhou, China; Central Texas Veterans Health Care System, Temple, Texas, USA

**Keywords:** peripheral blood, mNGS, mucorales, invasive mucormycosis

## Abstract

This study aims to assess the usefulness of peripheral blood metagenomic next-generation sequencing (mNGS) in diagnosing invasive mucormycosis (IM). We conducted a retrospective analysis of clinical data from 73 patients with mucorales sequences detected by peripheral blood mNGS at the First Affiliated Hospital, Zhejiang University School of Medicine, between February 2021 and December 2022. We examined data on risk factors, clinical symptoms, imaging characteristics, pathogenic findings, clinical diagnoses, and treatment outcomes. Among the 73 patients with mucorales sequences detected by peripheral blood mNGS, 7 cases were confirmed, 12 were probable, 34 were possible, and 20 were false positives, based on the diagnostic criteria for IM. Among the 34 possible cases, patients exhibited risk factors, clinical symptoms, imaging features, and prognoses consistent with IM. In 7 out of the 12 probable cases, the same mucorale nucleic acid was found in peripheral blood mNGS samples collected at an average interval of 8.1 days. In the remaining 20 patients, mucorales nucleic acid was detected in peripheral blood mNGS, but they lacked risk factors or clinical features of mucorales infection and lacked other pathological evidence, leading to a judgment of false positive. The positive predictive value of peripheral blood mNGS for diagnosing invasive mucormycosis in the cases studied was 72.6%. Peripheral blood mNGS testing provides valuable insights for diagnosing invasive mucormycosis. However, it should be used in conjunction with risk factors, clinical features, and other pathogenic test results to make a comprehensive assessment.

**IMPORTANCE:**

Given the high fatality rates, prompt and accurate identification of the fungal culprit is crucial, emphasizing the need for invasive mucormycosis. Unfortunately, mucormycosis lacks definitive biomarkers, depending primarily on smears, cultures, or pathology, all necessitating invasive specimen collection from the infection site. However, obtaining valid specimens early in critically ill patients poses substantial risks and challenges. Whether peripheral blood metagenomic next-generation sequencing (mNGS) can enhance early mucormycosis diagnosis, especially when direct specimen collection from the infection site is challenging, is warranted. This is a large-scale clinical study conducted to evaluate the utility and clinical impact of mNGS of peripheral blood for the diagnosis of invasive mucormycosis. We believe our study provided both novelty in translational medicine and a great value for the medical community to understand the strengths and limitations of mNGS of peripheral blood as a new diagnostic tool for the diagnosis and management of invasive mucormycosis.

## INTRODUCTION

Mucormycosis is a rare fungal disease caused by mucorales, primarily affecting immunocompromised individuals, including those with hematologic malignancies, hematopoietic stem cell transplantation, organ transplantation, immunosuppressive medication use like glucocorticoids, diabetes, burns, severe trauma, and drowning (1, 2). The order *Mucorales* encompasses 55 genera and 261 species, with 38 species being pathogenic to humans. Notably, *Rhizopus arrhizus* is the most common pathogenic genus worldwide, followed by *Mucor* and *Rhizomucor*. *Apophysomyces* and *Cunninghamella* are less common (2 – 5). Mucormycosis presents in various clinical forms, including naso-orbital cerebral, pulmonary, gastrointestinal, cutaneous, disseminated, and mixed. The diagnosis typically relies on tissue pathology or tissue culture from the infection site (6). These infections often involve vascular invasion, leading to thrombosis and tissue necrosis (7). In immunocompromised individuals, disseminated mucormycosis carries a staggering 95% mortality rate (8), and patients with hematologic malignancies and hematopoietic stem cell transplantation can face mortality rates of 65%–90% when infected (1). Given the high fatality rates, prompt and accurate identification of the fungal culprit is crucial, emphasizing the need for swift diagnosis and effective treatment. Unfortunately, mucormycosis lacks definitive biomarkers, depending primarily on smears, cultures, or pathology, all necessitating invasive specimen collection from the infection site. However, obtaining valid specimens early in critically ill patients poses substantial risks and challenges. Metagenomic next-generation sequencing (mNGS) offers a wide-ranging and highly sensitive pathogen detection method capable of accurately identifying fungal species and even subspecies. Given mucorales’ strong vascular invasion tendencies, investigating whether peripheral blood mNGS can enhance early mucormycosis diagnosis, especially when direct specimen collection from the infection site is challenging, is warranted. Here, we retrospectively analyzed clinical data from 73 patients with mucorales detected by peripheral blood mNGS at the First Affiliated Hospital, Zhejiang University School of Medicine, between February 2021 and December 2022, as detailed below.

## MATERIALS AND METHODS

### Study subjects

Patients who underwent peripheral blood mNGS testing at the First Affiliated Hospital, Zhejiang University School of Medicine, between February 2021 and December 2022, and had *Mucorales* pathogens detected were included. The comprehensive clinical diagnosis of invasive *Mucorales* infections or non-infections was determined by three senior specialists in respiratory, infectious diseases, and imaging. This diagnosis was made following discussions with the patients’ healthcare team, considering clinical symptoms, laboratory results, imaging, microbiology, pathological examinations, treatments, and prognoses. Cases with incomplete clinical data, individuals under 18 years of age, and pregnant women were excluded.

### Relevant definitions

The diagnosis of invasive mucormycosis (IM) is categorized into confirmed, probable, and possible diagnoses. In this study, cases were classified based on at least one blood mNGS detection of *Mucorales*, following the guidelines for invasive fungal diseases by the European Confederation of Medical Mycology and the Activities of the Intensive Care Unit Education and Research Consortium Working Group (9). The confirmed diagnostic criteria involve surgical or biopsy histopathology at the infection site that is aligned with *Mucorales* infections. A probable diagnosis is required to adhere to host factors, clinical signs or symptoms, and microbiological criteria for *Mucorales* infections. The microbiological criteria include *Mucorales* detection through microscopic examination of smears or culture from the infection site and detection of the same *Mucorales* pathogen through mNGS on more than two occasions (including blood or specimens from the infection site). A possible diagnosis necessitates the alignment with host factors and clinical features of *Mucorales* infection. Host factors encompass hematologic malignancy, agranulocytosis, diabetes mellitus with or without ketoacidosis, chemotherapy or immunotherapy, solid organ transplantation (SOT), hematopoietic stem cell transplantation (HSCT), granulocyte deficiency, prolonged glucocorticoid use (more than 3 weeks at an average lowest dose of prednisone equivalent to 0.3 mg/kg/day), trauma, and use of broad-spectrum antibacterial drugs and/or voriconazole (10). The clinical criteria for diagnosing pulmonary mucormycosis involve characteristic imaging findings such as the reverse halo sign, non-characteristic imaging presentations like lobar and segmental solid lesions, single or multiple nodules and masses, or ground-glass lesions, followed by more extensive imaging manifestations and vascular invasion leading to necrosis.

The included patients were categorized as confirmed, probable, or possible invasive mucormycosis based on the diagnostic criteria. In cases where clinical evidence and follow-up results do not support the diagnosis of invasive mucormycosis, peripheral blood mNGS results for *Mucorales* nucleic acid are considered to be false positives.

### mNGS testing

Blood samples were collected and immediately transported to the laboratory for mNGS testing within 1 hour after collection. The detailed methods regarding the wet lab and bioinformatics had been described previously (11). Most mNGS results returned within 24–48 hours.

### Data collection

Demographic information, risk factors, underlying diseases, use of immunosuppressive drugs or chemotherapeutic agents within 1 month before the onset of mucormycosis, clinical symptoms and laboratory tests, imaging data, and treatment and prognosis of the enrolled patients were collected.

### Comparison of clinical characteristics between patients with invasive mucormycosis and false-positive group

The clinical data of patients with invasive mucormycosis were statistically compared with those of the false-positive group to explore potential differences in the general condition, risk factors, clinical manifestations, and prognosis.

### Statistical analysis

Data were analyzed using SPSS 26.0 software (IBM Corp., Chicago, IL, USA). Continuous variables that followed a normal distribution were expressed as the mean ± standard deviation (*x* ± *s*), and independent-sample *t*-tests were utilized to compare measured information between groups. For continuous variables that did not conform to a normal distribution, median and interquartile range were used, and Mann–Whitney *U*-tests were employed for comparisons. Count data were compared using the chi-squared test or Fisher exact test. A *P*-value <0.05 was considered statistically significant.

## RESULTS

### General information of enrolled patients

**Fig 1:**
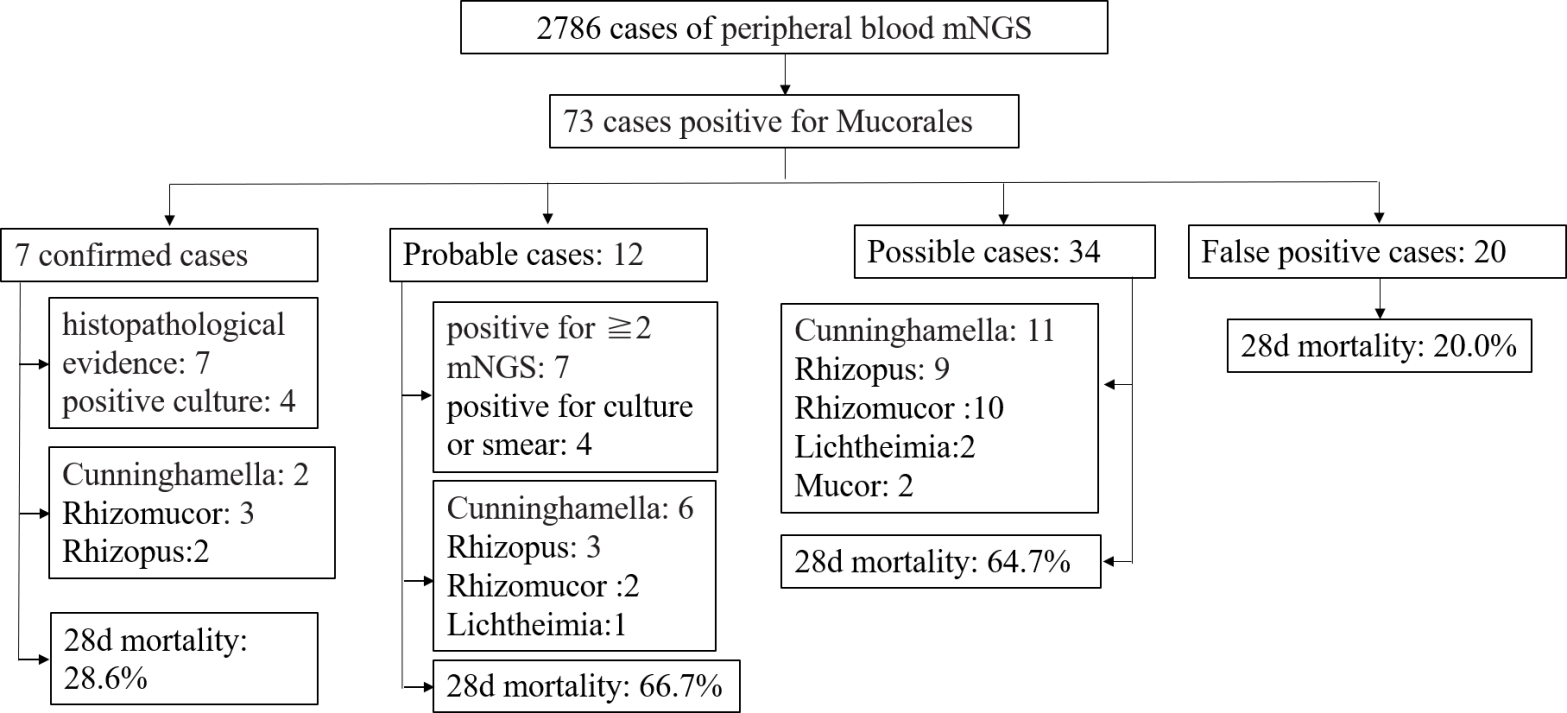
Overall clinical and pathogen diagnosis of the recruited patients.

A total of 2,786 patients underwent peripheral blood mNGS testing at the First Affiliated Hospital, Zhejiang University School of Medicine, between February 2021 and December 2022. Out of these, 73 cases tested positive for *Mucorales*, comprising 46 (63.0%) males and 27 (37.0%) females, with ages ranging from 19 to 87 years (mean age 53.8 ± 15.3 years). The most prevalent underlying disease was hematologic malignancy in 51 cases (69.9%), including 35 cases (47.9%) of leukemia, 9 cases (12.3%) of lymphoma, 6 cases (8.2%) of myelodysplastic syndrome, and 1 case (1.4%) of multiple myeloma. This was followed by 16 cases (21.9%) of HSCT, 7 cases (9.6%) of SOT, 7 cases (9.9%) of diabetes mellitus, 3 cases (4.1%) of solid tumors, and 1 case (1.4%) of drowning (Fig. 1).

### Confirmed cases

Histopathologically confirmed IM was observed in seven patients, including five males, with ages ranging from 28 to 69 years (mean age 52.3 ± 14.5 years). Besides the pathological and peripheral blood mNGS evidence, four patients had both positive cultures and specimens from the infection site that tested positive for *Mucorales*. Two deaths were reported within 28 days (Table 1).

**TABLE 1:** Clinical data of seven patients with confirmed diagnosis of invasive mucormycosis

Case	Underlying disease	Clinical manifestation	Radiographic findings	Blood mNGS	Infection site	Histopathologic diagnosis	mNGS results of infected site specimen	Smear or culture results of specimens from infected sites
1	Leukemia	Fever, expectoration, hemoptysis	Reversed halo sign	*Cunninghamella*	Lung	Surgical resection specimen of the upper lobe of the left lung and parabronchial lymph nodes	NA	NA
2	Lymphoma	Fever	Reversed halo sign	*Rhizomucor*	Lung, spleen, diaphragm	Spleen and left diaphragm	NA	Sputum immunofluorescence: *Mucorales* mycelium
3	Leukemia	Fever	Reversed halo sign	*Rhizomucor*	Lung	Surgical resection specimen of the right upper lung	BALF: *Rhizopus*	NA
4	Lymphoma	Fever, back pain	Massive	*Rhizomucor*	Liver	Surgical resection specimen of the right kidney	NA	NA
5	Diabetes mellitus, liver transplantation	Fever, gangrene of left upper arm wound, local purulent exudation with foul odor	NA	*Rhizopus*	Skin	Specimen of surgical debridement of the left upper arm	*Rhizopus*	Skin pus culture: *Rhizopus* spp.
6	Hepatocellular carcinoma and liver transplantation	Fever, consciousness disorders, dyspnea, scattered skin ulcers on limbs and trunk	Massive, pleural effusion, skin lesions	*Cunninghamella*	Lung, brain, skin	Bronchial mucosal biopsy	BALF:*Cunninghamella*	Sputum culture: *Cunninghamella*
7	Leukemia, breast cancer	Fever, dyspnea	Oropharyngeal cellulitis	*Rhizopus*	Pharyngeal cellulitis	Surgical specimen of necrotic tissue from pharyngeal tissue	*Rhizopus*	*Rhizopus*

### Probable diagnosed patients

There were 12 patients, including seven males, aged 34–65 years (mean age 49.1 ± 10.4 years), all diagnosed as probable cases of invasive pulmonary mucormycosis. Underlying diseases included leukemia in five cases, lymphoma in three cases, hematopoietic stem cell transplantation in two cases, post-liver transplantation in two cases, and diabetes mellitus in one case. In eight of these cases, the same *Mucorales* species were detected by mNGS in peripheral blood on two to three occasions, with intervals ranging from 1 to 19 days (mean interval 8.1 ± 6.2 days). Five cases had positive cultures or smears. The identified species included six cases of *Cunninghamella* sp., two cases of *Rhizopus microsporus*, one case of *Rhizopus oryzae*, one case of *Rhizomucor miehei*, one case of *Rhizomucor pusillus*, and one case of *Lichtheimia ramosa*. All lung CT scans showed imaging findings consistent with mucorales infection, such as nodular shadows, clumped shadows, anticorona, pleural effusion, and solid changes. Despite aggressive treatment, eight of these cases died within 28 days.

### Possible diagnosed patients

Thirty-four patients, including 22 males (64.7%), exhibited both risk factors and clinical features consistent with mucorales infections, although *Mucorales* nucleic acid sequences were detected by blood mNGS on only one occasion. Among them, 22 (64.7%) patients did not survive beyond 28 days. Clinical characteristics are presented in Table 2.

**TABLE 2:** Clinical data of 34 patients with possible diagnosis of invasive trichinosis

Demographic characteristics	Male	22 (64.7%)
	Age (years)	53.7 ± 15.8
Risk factor	Leukemia	17 (50%)
	Hematopoietic stem cell transplantation	10 (29.4%)
	Myelodysplastic syndrome	5 (14.7%)
	Lymphomas	3 (8.8%)
	Liver transplantation	2 (5.9%)
	Diabetes	2 (5.9%)
	Glucocorticoid usage	2 (5.9%)
	Hemophagocytic syndrome	2 (5.9%)
	Alcoholism with impaired consciousness	2 (5.9%)
	Digestive tract perforation	1 (2.9%)
	Drown	1 (2.9%)
Pulmonary imaging in cases of pulmonary infection	Mass	13 (38.2%)
	Reversed halo sign	12 (35.3%)
	Nodule	7 (20.6%)
	Patchy shadow	5 (14.7%)
	Pleural effusion	9 (26.5%)
	Solidation	4 (11.8%)
	Exudate shadow	1 (2.9%)
	Cavity	1 (2.9%)
	Ground-glass shadow	1 (2.9%)
Infectious site	Lungs	32 (94.1%)
	Disseminated infection	3 (8.8%)
	Skin and soft tissue	1 (2.9%)
	Gastrointestinal tract	1 (2.9%)
*Mucorales* species belonging to the genus *Mucorales*	*Cunninghamella* sp.	11 (32.4%)
	*Rhizomucor* sp.	10 (29.4%)
	*Rhizopus* sp.	9 (26.5%)
	*Mucor* sp.	2 (5.9%)
	*Lichtheimia* sp.	2 (5.9%)

### False-positive cases

There were 20 cases, including 12 males (60.0%), with ages ranging from 20 to 87 years (mean age 56.7 ± 16.7 years) among the false-positive patients. Underlying diseases included 10 cases of leukemia, 4 cases of HSCT, 3 cases of diabetes mellitus, and 1 case each of lymphoma, myelodysplastic syndromes, multiple myeloma, colon cancer, multiple systemic atrophy, and aortic coarctation. Among these 20 cases of mucormycosis, 12 cases were caused by *Rhizopus* sp., 3 cases by *Mucor* sp., 3 cases by *Cunninghamella* sp., and 2 cases by *Rhizomucor* sp. The 28-day mortality rate in this group was 20.0%.

### Comparison of clinical characteristics between patients with invasive *Mucorales* and false-positive patients

When comparing clinical data between the 53 patients with invasive mucormycosis and the 20 false-positive patients, it was observed that the presence of the reverse halo sign and mass shadow was more common in lung CT imaging among the infected group. Patients in the infected group had lower leukocyte counts, a higher number of mucorales reads detected by mNGS, and a poorer prognosis. Peripheral blood mNGS detected that *Cunninghamella* sp. and *Rhizopus* sp. were significantly more likely to indicate infection rather than contamination, while the detection of other *Mucorales* species required more cautious assessment to distinguish between infection and contamination (Table 3).

**TABLE 3:** Comparison of clinical characteristics of patients with true- and false-positive peripheral blood mNGS Mucorales

		Total (*n* = 73)	Infection group (*n* = 53)	False-positive group (*n* = 20)	*P*-value
Male		46 (63.0%)	34 (64.2%)	12 (60.0%)	0.743
Age (years)		57 (47, 64)	55.0 (43.5, 62.0)	56.7 ± 16.7	0.120
Underlying diseases	Hematologic malignancy	51 (69.9%)	38 (71.7%)	13 (65.0%)	0.578
	Hematopoietic stem cell transplantation	16 (21.9%)	12 (22.6%)	4 (20.0%)	>0.999
	Solid organ transplantation	7 (9.6%)	6 (11.3%)	1 (5.0%)	0.710
	Solid tumor	3 (4.1%)	2 (3.8%)	1 (5.0%)	>0.999
	Diabetes	7 (9.6%)	4 (7.5%)	3 (15.0%)	0.604
Lung CT imaging	Reversed halo sign	17 (23.3%)	17 (32.1%)	0	0.010
	Nodule	13 (17.8%)	9 (17%)	4 (20.0%)	>0.999
	Patchy shadow	9 (12.3%)	7 (13.2%)	2 (10.0%)	>0.999
	Mass	22 (30.1%)	22 (41.5%)	0	0.01
	Pleural effusion	19 (26.0%)	14 (26.4%)	5 (25.0%)	0.902
	Exudate shadow	2 (2.7%)	1 (1.9%)	1 (5.0%)	0.476
	Solidation	9 (12.3%)	6 (11.3%)	3 (15.0%)	0.978
	Ground-glass shadow	1 (1.4%)	1 (1.9%)	0	>0.999
	Cavity	2 (2.7%)	1 (1.9%)	1 (5.0%)	0.476
Laboratory tests	WBC (×10^9^/L)	1.3 (0.3, 5.9)	0.7 (0.2, 3.4)	4.2 (0.5, 8.2)	0.122
	WBC ≤ 1 × 10^9^/L	32 (43.8%)	27 (50.9%)	5 (25%)	0.046
	NE (×10^9^/L)	0.9 (0.1, 4.8)	0.4 (0.04, 3.18)	2.2 (0.1, 6.5)	0.173
	PLT (×10^9^/L)	26 (11, 44)	18 (10.5, 40.5)	42.5 (16.3, 132.3)	0.014
	Albumin (g/L)	31.8 ± 5.0	31.3 (28.5, 33.1)	32.3 ± 4.9	0.327
	CRP (mg/L)	72.7 (39.5, 137.5)	79.8 (44.1, 174)	54.7 (26.9, 107.2)	0.327
	PCT (ng/mL)	0.6 (0.2, 2.6)	0.6 (0.3, 2.0)	0.3 (0.1, 6.9)	0.440
	Read per million (RPM) of *Mucorales* in mNGS	21 (5.5, 224.5)	53 (10, 420)	4.5 (3, 23)	0.001
*Mucorales* species	*Rhizopus* sp.	26 (35.6%)	14 (58.3%)	12 (60.0%)	0.008
	*Cunninghamella* sp.	22 (30.1%)	19 (86.4%)	3 (15.0%)	0.083
	*Rhizomucor* sp.	17 (23.3%)	15 (88.2%)	2 (10.0%)	0.180
	*Mucor* sp.	5 (6.8%)	2 (40.0%)	3 (15%)	0.240
	*Lichtheimia* sp.	3 (4.1%)	3	0	0.557
Clinical manifestation	Fever	69 (94.5%)	50 (94.3%)	19 (95.0%)	>0.999
	Chest distress	26 (35.6%)	21 (39.6%)	5 (25.0%)	0.245
	Dyspnea	25 (34.2%)	21 (39.6%)	4 (20.0%)	0.115
	Cough	28 (38.4%)	19 (35.8%)	9 (45.0%)	0.473
	Expectoration	21 (28.8%)	16 (30.2%)	5 (25.0%)	0.662
	Hemoptysis	8 (11.0%)	7 (13.2%)	1 (5.0%)	0.561
Mortality	14 days	29 (39.7%)	27 (50.9%)	2 (10.0%)	0.001
	28 days	36 (49.3%)	32 (61.4%)	4 (20.0%)	0.002

## DISCUSSION

Mucorales are fungi that can become pathogenic under certain conditions. They are commonly found in soil, plants, and decaying organic matter. Infections can occur through the inhalation of spores into the nostrils, oropharynx, or lungs, ingestion of contaminated food or water, or inoculation into broken skin or wounds. Once a person is infected with mucorales, the disease progresses rapidly, exhibits high invasiveness, and carries a significant risk of mortality. Some research has suggested that the patient’s underlying health condition is linked to the site of infection, with pulmonary mucormycosis being more prevalent in individuals with hematologic malignancies and neutropenia (4). In this study, pulmonary involvement was observed in 35 cases within the group of patients with invasive *Mucorales* infections, accounting for 66% of the cases.

The non-specific clinical signs and symptoms of invasive mucormycosis pose challenges for early diagnosis. Nevertheless, timely and accurate diagnosis is crucial to enhance prognosis, constituting the primary objective of ongoing research. The diagnosis of mucormycosis relies on direct microscopic examination, culture, and histopathology. However, obtaining samples from the infection site, particularly in critically ill patients, is challenging and yields low sensitivity. The advantage of blood samples is that they are easy to obtain and almost non-invasive. Blood cultures yield a very low positive rate, carry a high risk of contamination, and offer limited diagnostic value. The predominant issue we currently face is the absence of non-invasive, rapid, and dependable diagnostic methods. Creating a culture-independent biomarker for early invasive mucormycosis diagnosis is an unfulfilled clinical requirement in modern mycology. Molecular diagnostic methods like PCR assays and mNGS are known for their speed and high sensitivity. These techniques are highly suitable for patients with complex and critical infections who cannot undergo invasive tests such as tracheoscopy, puncture biopsy, or surgery. The International Fungal PCR Initiative is striving to integrate fungal PCR diagnostics into the EORTC/MSG guidelines. It has successfully incorporated *Aspergillus* PCR diagnostics into these guidelines for *Aspergillus*. Although mucorales PCR has not yet been included in these standards due to the lack of standardization or test availability, it has significantly improved mucormycosis diagnosis in recent years. Fluorescence *in situ* hybridization methods for promptly identifying *Mucorales* from formalin-fixed, paraffin-embedded tissues, along with real-time quantitative PCR systems for precise genus- and species-level identification, have been developed to accurately detect pathogenic *Mucorales* in tissues (12). A French study, conducted at multiple centers, assessed the usefulness of qPCR in diagnosing invasive trichothecosis through serum samples. The study revealed a diagnostic sensitivity of 85%, a specificity of approximately 90%, and a negative predictive value of roughly 98% (13). Consequently, *Mucorales* PCR can aid in detecting circulating DNA in peripheral blood for clinical mucormycosis diagnosis and ongoing treatment monitoring. It was observed that serum contained a notably high load of circulating *Mucorales* DNA, likely attributed to *Mucorales* infection’s vascular invasiveness. Hence, blood samples are suitable for screening and monitoring treatment in high-risk patients (14). mNGS stands as a cutting-edge pathogenicity assessment technology, enabling unbiased detection of numerous fungi, viruses, bacteria, and rare pathogens. Pathogens can be identified in serum, tissue, and secretion samples (15). This method permits the identification at both the genus and species levels (16). In challenging and severe cases suspected of invasive mucormycosis, blood and alveolar lavage fluid can be collected for mNGS testing (6).

Currently, quantitative PCR conducted on serum or tissue serves as a valuable molecular diagnostic technique for *Mucorales* spp. (17). Blood mNGS can identify invasive mold infections in blood samples, offering the potential for early diagnosis. The relatively high positive predictive value (72.6%) of *Mucorales* infections detected by blood mNGS in our cases may be due to the enrollment of a substantial number of high-risk individuals. However, as a retrospective study, it is subject to inherent limitations like selection bias. Among the 73 patients included in this study, 51 (69.9%) had hematologic malignancies, and only 7 received a confirmed diagnosis through histopathology. Additionally, the current study did not perform a sensitivity and specificity assessment of the blood mNGS test for diagnosing invasive mucormycosis.

Mucorales are fungi commonly found in the natural environment, including laboratories and clinical indoor settings, making false-positive results in clinical blood samples for mNGS testing unavoidable, from sampling to sample processing. Clinical diagnosis still requires a comprehensive assessment, combining risk factors, clinical characteristics, and other pathogen test results. This study observed that the RPM of *Mucorales* sequences detected by blood mNGS was significantly lower in the false-positive patients compared to the infected group, aiding clinicians in effectively interpreting *Mucorales* results obtained through blood mNGS.

The results of this study have reference value for clinical practice. Our study is a single-center retrospective study with a small number of enrolled cases. These limitations make us look forward to a well-designed multicenter prospective study to further evaluate the value of peripheral blood mNGS technology in the diagnosis of invasive mucormycosis and gain a more comprehensive and in-depth understanding of its advantages and disadvantages.

### Conclusion

Peripheral blood mNGS demonstrates a strong positive predictive value for diagnosing invasive mucormycosis, particularly among patients presenting with the following characteristics: agranulocytosis, lung CT imaging showing pertinent features, and a high RPM of *Rhizopus* sp. or *Cunninghamella* sp., among others.
